# Understanding collaborative working between GPs and community pharmacists: systematic review and thematic synthesis of qualitative studies

**DOI:** 10.3399/BJGPO.2024.0203

**Published:** 2025-12-19

**Authors:** Grace Kng Li Lin, Aleema Sardar, David N Blane

**Affiliations:** 1 Watsons Singapore, Singapore, Singapore; 2 University of Glasgow, Glasgow, UK; 3 General Practice & Primary Care, School of Health & Wellbeing, University of Glasgow, Glasgow, UK

**Keywords:** primary health care, qualitative research, systematic review

## Abstract

**Background:**

Collaborative working between GPs and community pharmacists (CPs) has led to improvements in management of long-term conditions, and has strong policy backing, yet such joint working remains patchy and underdeveloped. Understanding the perspectives of GPs and CPs regarding collaboration provides insights for more sustainable collaborative practices.

**Aim:**

To understand the perspectives of GPs and CPs concerning collaborative care practices, and to develop a framework of factors that influence this collaborative working relationship.

**Design & setting:**

A systematic review of qualitative studies.

**Method:**

Five databases were searched from inception to the 22 April 2023 for qualitative studies exploring GP and CP views on collaborative care services. Articles were independently screened by two reviewers at title, abstract, and full-text levels. Data extracted from eligible studies were analysed and synthesised thematically.

**Results:**

Thirty-six studies met the inclusion criteria. The following four main themes were identified: (1) how pre-existing relationships influence mutual understanding of roles; (2) the impact of co-location and communication on relationship building; (3) analysis of perceived costs and benefits of collaborative care; and (4) unequal motivation to collaborate.

**Conclusion:**

A complex interplay of factors influences GP and CP collaborative working, including pre-existing relationships, communication and mutual understanding, and the balance of costs and benefits to further develop these relationships. When planning future collaborative care practices, stakeholders must take the time and initiative to elucidate and understand these factors within their own unique healthcare setting to form lasting working relationships.

## How this fits in

Previous research has quantified the clinical and economic benefits of collaborative care services between GPs and CPs, but little is known about how both professional groups perceive the change in roles and expectations, which collaborative care entails. These perspectives influence motivation for collaboration, which in turns influences its sustainability and success. This article identifies key themes that influence the development and continuation of collaborative working relationships.

## Introduction

In recent years, there have been policy and practice drivers in several high-income countries for GPs and community pharmacists (CPs) to work more closely together to improve patient care.^1–9^ There is some evidence that strong collaborative relationships lead to improved patient outcomes in conditions such as diabetes and hypertension.^10–12^ However, it is clear that — in practice — collaboration between GPs and CPs remains challenging.^13,14^ Barriers to collaboration include a lack of mutual understanding of roles, poor communication, traditional medical hierarchies, and practical constraints such as geographic distance and CPs lacking access to patients’ medical records.^15–17^

Previous research has attempted to identify factors that shape collaborative working.^10,18,19^ For instance, the Collaborative Working Relationship model characterises factors at the individual (practitioner), interpersonal, and institutional (practice) levels that influence successful collaboration.^20^ Other studies suggest that key drivers of successful collaboration include trust (typically pharmacists gaining physicians’ trust) and clarity of roles within the collaborative working relationship.^10,21^ Similarly, a more recent Delphi consensus study highlighted the importance of good communication and mutual role understanding and outlined a range of practical actions that could improve collaboration.^22^

However, these models remain largely theoretical in nature. They may not accurately reflect the complex interplay of factors found in current healthcare systems and do not focus on practical solutions to support collaborative working practices. Hence, the aim of this study was to synthesise qualitative studies of the views and experiences of GPs and CPs concerning collaborative care practices, in order to develop a framework of factors that influence this collaborative working relationship, based on empirical evidence.

## Method

This systematic review of qualitative studies was conducted according to a pre-specified protocol and is reported according to the Preferred Reporting Items for Systematic reviews and Meta-Analyses (PRISMA) and Enhancing transparency in reporting the synthesis of qualitative research (ENTREQ) guidelines.^23^

### Search strategy and eligibility

We searched five databases (MEDLINE, Embase, PsychInfo, CINAHL, Web of Science) and Google Scholar from inception to 23 April 2023. The search strategy was developed in collaboration with an information specialist and is shown in Table 1 (full search terms in Supplementary file S1).^24,25^

**Table 1.** Search strategy

Sample	General practice or general practitioner Family practice OR family practitioner Primary physician OR family physician Family medicine Primary health care OR primary care Community pharmacist OR community pharmacy Retail pharmacist OR retail pharmacy Pharmacist
Phenomenon of interest	Collaborate OR Collaborative (Relationship OR Practice*) eg, Collaborative care practice or collaborative work relationship Cooperate OR Cooperative (Relationship OR Practice* OR behavio?r) Interprofessional Relation*/Relationship* Intersectoral collaboration Multidisciplinary Care Integrated Service* Joint practi?e*
Design	questionnaire* OR survey* OR interview* OR focus group* OR case stud* OR case report* OR observ*
Evaluation	Opinion OR feedback OR view OR Viewpoint OR impression* OR perspective* OR perception OR belief* OR believe* OR attitude* OR expectation OR assumption Role Perception
Research type	qualitative OR mixed method*

Articles were eligible if they included qualitative data on the views and experiences of either GPs or CPs concerning collaborative care practices in a community (primary care) setting. Only peer-reviewed articles in English were included.

We defined collaborative care practices between GPs and CPs as a cooperative and coordinated approach in which both healthcare professionals work together to optimise patient care. This may involve sharing knowledge and expertise, as well as jointly making decisions regarding patient therapy. It can involve activities such as medication optimisation and chronic disease management. Healthcare professionals may collaborate in the same practice (co-location), or within separate practice settings.

References were imported into the systematic review tool DistillerSR software, which was used to help manage the database of reference and remove duplication. Following which title, abstract, and full-article screening were performed independently, without the assistance of AI tools, by AL and GK with any conflicts resolved by DB.

### Data extraction and analysis

Data extraction was done using a pre-piloted form (Supplementary Table 1) on DistillerSR with the study characteristics documented and recorded in Supplementary Table S2. Analysis of the data were conducted using the standardised method described by Thomas and Harden.^26^ All articles were read at a minimum of three times. At each read-through, key concepts (for example, the cost of setting up collaborative practices) were identified through both direct quotes from study participants and the authors’ analysis of these quotes. These key concepts were subsequently grouped into overarching themes describing healthcare professionals’ perceptions of collaborative care practices (for example, which financial pressures influence collaboration). The research team discussed how these ‘descriptive themes’ related to the barriers and facilitators surrounding collaborative practices. The data were re-examined in light of these discussions until ’analytical themes’ were developed, which were sufficient to explain our ‘descriptive themes’.

### Quality appraisal

Studies were assessed for quality using the Critical Appraisal Skills Programme (CASP) checklist for qualitative studies.^27^ Studies were not excluded based on quality.

## Results

**Figure 1.**
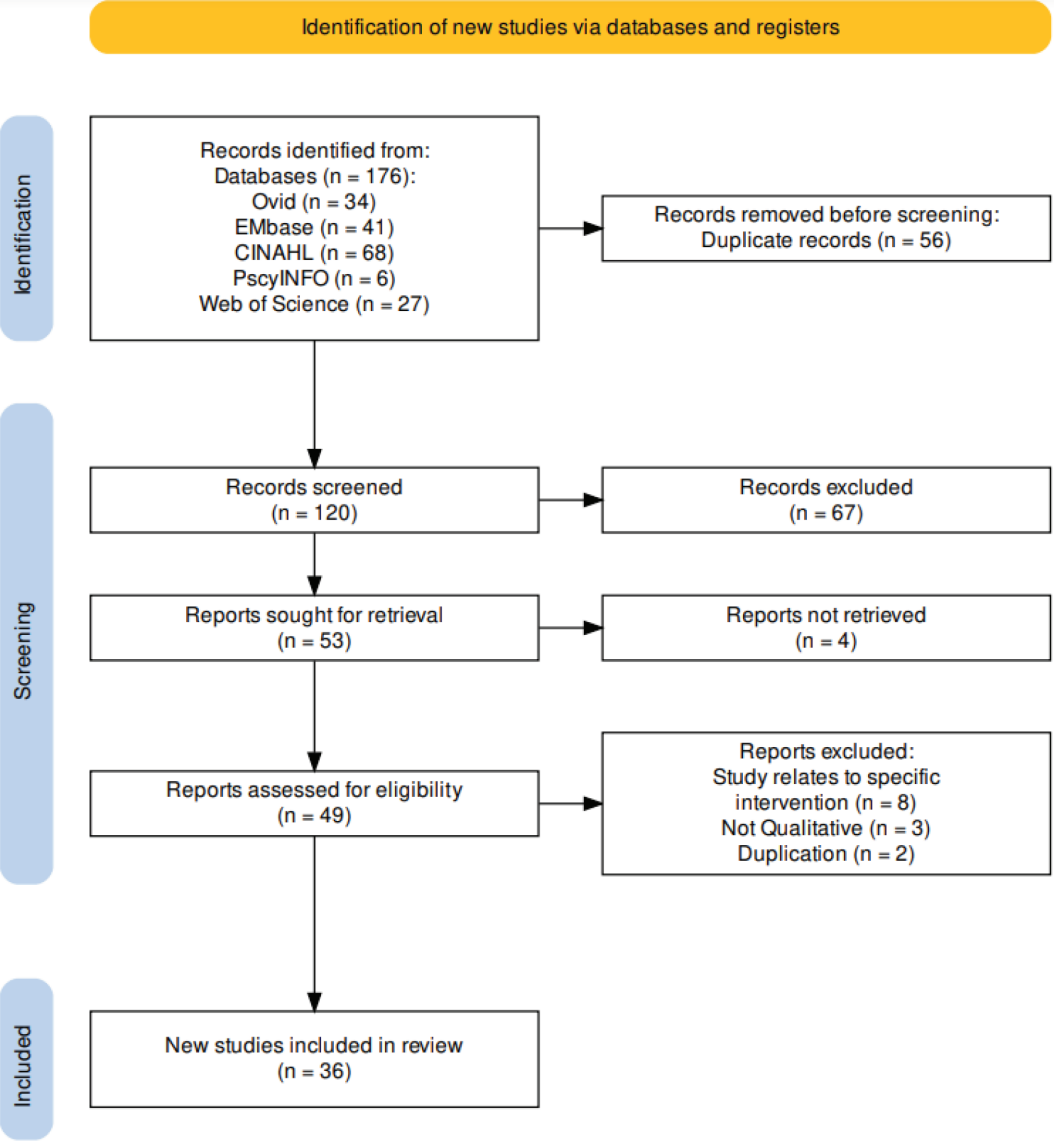
Preferred Reporting Items for Systematic reviews and Meta-Analyses (PRISMA) flow diagram

A total of 36 articles were included (see Figure 1 for PRISMA flow diagram). The 36 articles come from mainly high-income countries such as Australia (*n* = 9), the UK (*n* = 8), US (*n* = 7), Canada (*n* = 3), and several European countries (*n* = 7). There were two articles from Malaysia. Apart from Malaysia, all these countries practise dispensing separation. Many (for example, UK, Australia, and Canada) have a history of supporting collaboration between GPs and CPs, and some articles from these locations evaluate ongoing collaborative care projects.^11^ However, most articles evaluate perceptions of collaboration in settings where there was no prior collaborative experience (Supplementary Table 2).

The quality of most studies was deemed relatively high based on the CASP tool for qualitative research (Supplementary Table 3). However, nearly all studies did not document if the relationship between the researcher and the participants was taken into consideration. Several articles were also unclear about their research design and recruitment strategies. In some cases, this was because the article was part of a larger project, and relevant details were reported elsewhere.

### Thematic synthesis

**Figure 2.**
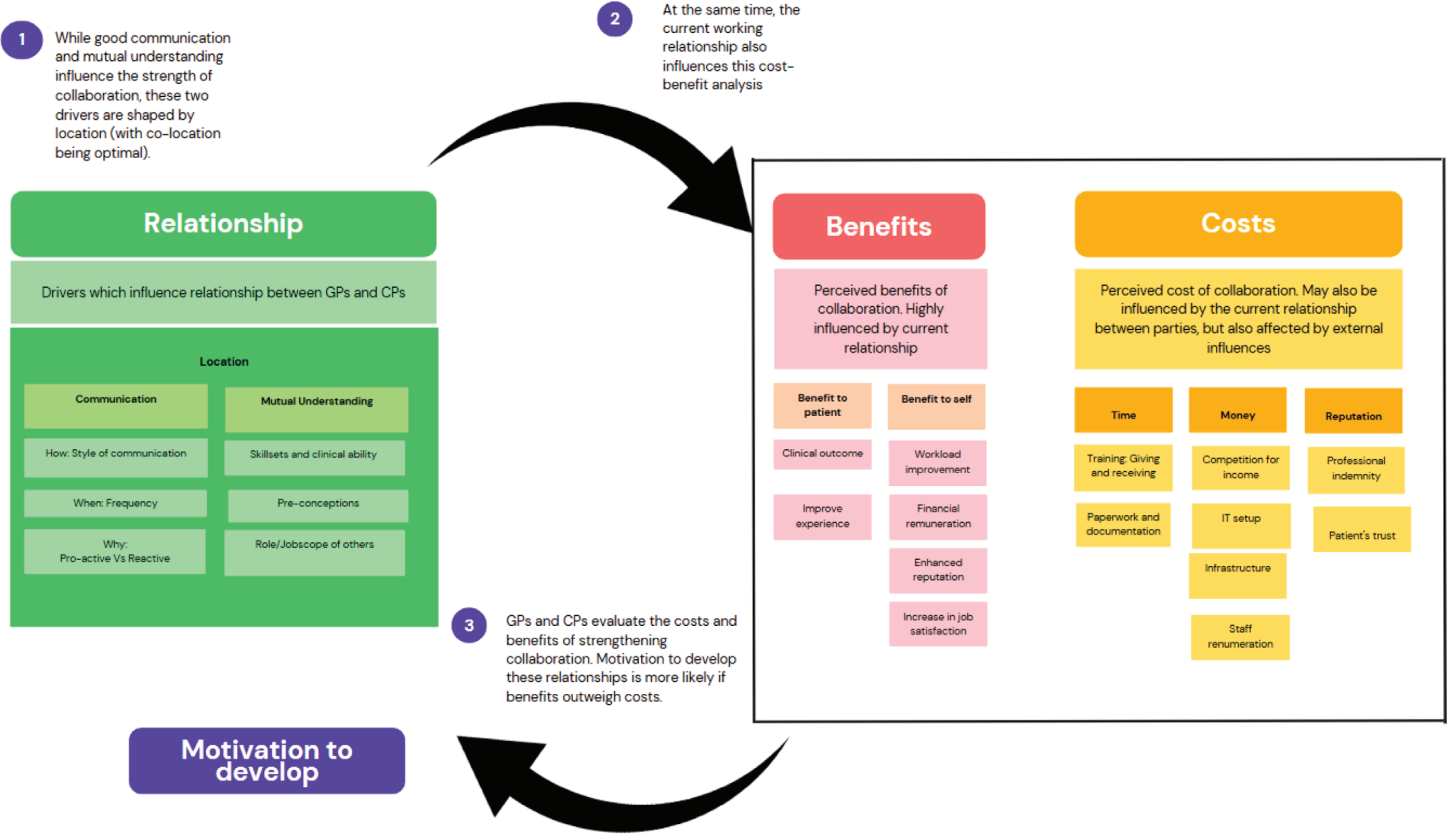
Summary of key findings

The following four main themes were identified: (1) how pre-existing relationships influence mutual understanding of roles; (2) the impact of co-location and communication on relationship building; (3) perceived costs and benefits of collaborative care; and (4) unequal motivation to collaborate. Each theme will be described in turn, with illustrative quotes from included studies identified by professional role (GP or CP). An illustration of key findings is presented in Figure 2.

### How pre-existing relationships influence mutual understanding of roles

Multiple studies highlighted that a key barrier to collaboration is lack of GP awareness and recognition of a CP’s clinical skillset:^16,28–45^

*‘I’m probably not gonna actually advise someone to go to a community pharmacist weight management* [sic]*, because I just don’t know what they do and how useful it is.’* (GP, UK)^16^

However, lack of awareness is bi-directional and some CPs in included studies acknowledged that they do not have a good understanding of the GP role either:^46^

*‘*[M]*ost pharmacists actually don’t know what goes on in a GP surgery. They don’t know what the actual function of the GP is.’* (CP, UK)^47^

This lack of awareness appears related to a lack of interaction^36^ and can be self-perpetuating.^16^ However, even GPs with past collaborative experience, and good understanding of the CP role, do not always have confidence in the clinical abilities of CPs.^30,33–35,38,41,48^ To them, sustained, personalised, individual relationship building tends to trump ‘*quick fixes*’ such as joint training programmes.^48^

### The impact of co-location and communication on relationship building

#### Co-location

One way to facilitate sustained relationship building is through integrating the CP directly into the GP practice. Hence, several articles explored participants’ views on such co-location. While there were concerns about practicalities of co-location, both GPs and CPs were positive, reporting benefits such as more time for in-depth patient counselling.^33–38,40–42,47–52^

Co-location was also reported to improve communication between professionals, by improving frequency of contact and creating opportunities for ad hoc communication.^35,38,40,42,48,50,53^ Co-location also improved the transfer of patient information, overcoming a commonly cited barrier to collaboration, when practical and technological limitations hampered information sharing:^35,40,50,51,53,54^

*‘It gave us the opportunity to speak to those GPs that we would ordinarily speak to on the phone very briefly. It gave us an opportunity to speak a bit more in-depth with them. Which again, I think gained a bit of mutual respect, so we built that really good relationship with them.*’ (CP, Australia)^53^

Most importantly, co-location increased mutual understanding of roles, with improved awareness of skillsets and capabilities.^16,35,37,38,45,47,48,50,55^ Co-location overcame pre-conceived stereotypes of CPs as ‘shopkeepers’,^56^ improving GPs’ trust in CPs.^56^ CPs also felt more accepted by GPs and their practice staff through co-location.^35,37,38,40,47,51,55^

#### Communication

Several aspects of communication were felt to influence relationship building, including frequency, mode, and reason. First, more frequent communication was felt to improve working relationships:^16,28,29,32,36,38,40,44,50,53^

*‘There’s* [sic] *a few other doctors that we do chat on a regular basis to the point where they don’t want us to call them doctors or by their surname. We have got each other’s mobile phones* [numbers]*.’* (CP, Australia)^50^

Second, mode of communication (for example, face to face versus telephone) was considered important, with most studies describing a preference for face-to-face communication for discussing clinical issues and improving rapport.^16,28,30,33,36–38,44,48,50,52,53,55,57^

Finally, the reason for communication made a difference to collaborative relationships. Before collaborative care practices, CPs’ communications with GPs were mainly reactive in nature, concerning potential prescribing errors or communicating other medication issues (for example, a drug being unavailable). This type of communication was frequently described by GPs as disruptive and overly critical:^29,32–34,36,38,41,43–45,47,50,52,53,55,57^

*‘Probably 9 out of 10 of the communications that I have with* [retail] *pharmacists start with some sort of negative tone, “Do you really want to prescribe this medication?”’*(GP, US)^34^

CPs had negative experiences with reactive communication too, impacting the working relationships between GPs and CPs:^32,33,36,44,45,53,56,58^

*‘Sometimes we actually fear calling there, because we are scared of being snapped at. You know, we’ve sometimes had such bad experiences. Of course, we’ve also had really good experiences, but you know, if you have ten really good experiences and only one bad one it is the bad experience that sticks.’* (CP, Germany)^32^

Communication based on building relationships (for example, taking time to ask about friends and family)^55^ within a collaborative care model was felt to build trust and was viewed positively, in contrast to communication purely for work-related matters:^59^:

*‘Taking the time to ask about friends, family, working relationships so that you actually become a nice colleague before you actually start moving on to do nice work. So you are already developing those relationships rather than trying to go in and implement change straight away.’* (CP, UK)^55^ 

### Perceived costs and benefits of collaborative care

As well as good communication and mutual understanding, the perceived costs and benefits to collaborative care practice were also noted to influence the drive to proceed with collaboration. Perceived costs included time costs, monetary costs, and potential reputational costs.

#### Time costs

The time required to establish a collaborative care practice was often cited as a barrier:^16,29,31,35–37,42,44,52^

*‘... previous pilot projects failed, e.g., they required too much time, there were too many meetings, and finally no outcomes.’* (CP, Germany)^29^

Proactive communications typically require a greater investment in time as these interactions often take place outside working hours. The increased documentation associated with effective collaboration was perceived by some as an unwelcome increase in workload for both GPs and CPs.^31,59^

#### Monetary costs

Money may be required to set up collaboration through the establishment of stronger IT services, physical infrastructure,^29^ or staff reimbursement.^31^ Monetary costs also include lack of funding for collaboration. Both GPs and CPs cited insufficient remuneration as a tipping point in their decision not to collaborate:^16,29,34,35,38,41,42,45,47,48,56,57^

*‘We provide a service. We would love to be paid for the service provided you know ... But the only way we can continue to provide such a service is to sell product and our margins are getting cut and cut and cut ... The fact* [is] *that pharmacy is small business, and there’s no funding for that extra, so it comes out of profit.’* (CP, Australia)^56^

Some studies reported GPs’ concerns about loss of income through direct competition, particularly where proposed collaborative care services mirror services that GPs already provide.^16,30,34,41,42,45,48,56,57^ This was most pronounced where GPs and CPs already compete for the same source of income before collaboration, such as countries (for example, Malaysia) with no dispensing separation or countries where minor ailment care is the mainstay of both GPs and CPs (for example, UK, Australia, Germany, Spain):^41,45,59^

*‘We find that pharmacist are taking our patients from us and GPs are suffering basically to even get that amount of patients they once had. Where do the patients go, they go straight to the pharmacist.*’ (GP, Malaysia)^41^

#### Reputational costs

Both GPs and CPs feared reputational damage when embarking on collaborative services, in the eyes of their patients and each other. While some CPs with no prior experience in collaborative practice were concerned about jeopardising their relationship with their GPs by embarking on collaboration:^16^

*‘I think we shouldn’t tamper with it (the relationship with the GP) … if we look for something more, we will have problems.’* (CP, Spain)^33^

Other GPs expressed concern about being ‘*criticised*’ for their therapeutic decisions by CPs, worrying that this might affect patient trust:^29,31,36,38,40,43,44,48,53,60^

*‘The fear, that you appear unqualified in front of someone else.’* (GP, Germany)^44^

CPs in contrast did not share this concern as many already had the impression that they were held in low regard by GPs and patients:^29,45,49,55^

*‘They* [some GPs] *don’t have any opinion at all about community pharmacists. They think we have no role, they think we are shopkeepers that are useless and who are grasping for greater things ...’* (CP, Northern Ireland)^38^

This imbalance of *‘power*’ was a source of disquiet for some CPs, who perceived GPs to be territorial when declining to collaborate.^35,36,38,45,55,58,61^ GPs, on the other hand, perceived themselves as appropriately assuming primary responsibility for patient care.^36,57^

#### Benefits

Perceived benefits of collaboration could be divided into benefits to patients and the healthcare system, and benefits to practitioners themselves. Those with past experience of collaborative care were more likely to recognise benefits:^30,31,33,35,37,41,43,47–49,51,55^

*‘We have a source on tap of drug information, and I can see his role in checking medication being extended to a whole host of things ... he could run clinics in the building and he could run surgeries in theory.’* (GP, UK)^48^

Those with no prior collaborative experience, however, struggled to imagine how collaborations could be beneficial to them or their patients:^29,33,35,41,45,59^

*‘I think that there has to be a normal relationship and if we need something, there can be one-off contact but I don’t see the need for regular contact.’* (GP, Spain)^33^

This difference in perception between those with prior collaborative experiences and those without further highlights the importance of prior relationship building, through improving communication and awareness.

### Unequal motivation to collaborate

There was a mismatch between GP and CP perceptions of the benefits of collaboration, with each assuming the other would benefit more. For example, many GPs assumed that CPs would financially benefit more as patients are referred to them.^16,30,34,41,42,48,56,57^ This perception is more prevalent in GPs who view CPs as competitors rather than colleagues. In addition, GPs were less convinced of the clinical or workload related benefits of collaboration, although this often related to their lack of understanding of CPs’ roles and abilities:^29–31,33,37,38,40,41,60,62^

*‘I can check the* [medication] *effects and the other information right here on my own. I can assess interactions, contraindications and all that with the software I use. Those things have become a lot simpler due to the software.’* (GP, Germany)^32^

CPs, however, were less likely to perceive a financial benefit,^37,55^ and more likely to emphasise how their services would benefit GPs; for example, reducing workload and preventing errors.^47^ CPs who pursue collaboration also mention the enhanced reputation (from GPs and patients) and job satisfaction as a driving factor,^31,35,56^ a motivation not observed among GPs:

*‘We’ll be working with GPs and it’s quite nice and encouraging to hear it from a GP’s mouth … To hear that “actually we want you, we need you and there’s room for you guys ... ”’* (CP, UK)^47^

Overall, while CPs do perceive costs to collaborations, they were more enthusiastic about the benefits of collaborative care. This imbalance in perceived costs and benefits ultimately affects motivation to collaborate, perpetuating the cycle demonstrated in Figure 2.

## Discussion

### Summary

This systematic review of qualitative studies synthesised the views and experiences of GPs and CPs from 36 studies across 11 different countries. We found that collaborative care between GPs and CPs is shaped by pre-existing relationships and role awareness, with co-location and good communication increasing the likelihood of successful collaborations. GPs and CPs held a range of views on potential costs (time, money, reputation) and benefits (to patients, practices, and health systems) of collaboration. Trust was a key enabler of collaborative working. The motivation to initiate collaborative care practices was frequently one-sided, with CPs generally more enthusiastic than GPs.

### Strengths and limitations

Strengths of this systematic review include pre-registration on the PROSPERO database, a comprehensive search strategy, and robust screening and data extraction processes. The use of thematic synthesis allows us to draw our conclusions based on common elements among heterogenous studies from diverse, international settings.

A potential limitation is the applicability of findings in settings that do not practise dispensing separation. Of the 11 countries represented in included studies, only Malaysia does not practise dispensing separation. The lack of dispensing separation may increase the competition between private GPs and CPs as both parties compete directly for the same source of income through medication dispensing. This may lead to a deeper sense of mistrust, with some of the harshest statements of mistrust being reflected in the Malaysian study.^41^ However, without further data from countries in a similar setting, it would be difficult to extrapolate the impact of such competition on the collaborative working relationship.

### Comparison with existing literature

**Figure 3.**
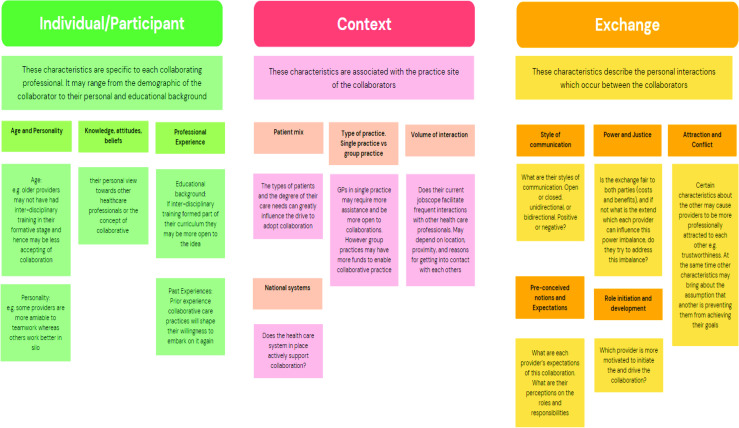
Adaptation of the Collaborative Working Relationship model by McDonough and Doucette^20^

Our results align with previous findings by McDonough and Doucette^20^ by demonstrating the importance of communication, trust, and inter-professional awareness of roles in building a collaborative working relationship between GPs and CPs. Their Collaborative Working Relationship model highlighted three characteristics — individual, contextual, and exchange — which influence the development of the GP–pharmacist relationship along a collaboration continuum (Figure 3).^20^ Individual characteristics involve the personal characteristics of the collaboration participants (for example, educational background), context characteristics are associated with their practice sites (for example, location), and exchange characteristics encompass the nature of interactions between each participant, underpinning the way practitioners perceive collaboration.^20,26^ Where there is no prior experience of collaboration, practitioners frequently rely on past impressions to build a cost-benefit analysis on whether to embark on collaboration.

The Collaborative Working Relationship model described how contextual characteristics, such as location, shape exchange characteristics and should be considered when developing collaborative working relationships. We found that co-location was considered to improve the development of trusting relationships. This may explain why stronger collaborative working relationships between physicians and pharmacists are more common in hospitals, where they have historically worked more closely together.

Previous studies,^10,21^ including those that did not meet the inclusion criteria of our review,^17,22^ have demonstrated that exchange characteristics are potentially the main drivers of the development of pharmacist–physician collaborations. Results of this review indicate that context characteristics (for example, co-location) and individual characteristics (for example, past experience with other healthcare professionals) also play a significant role in the development of exchange characteristics. All three must be carefully considered when developing strong working relationships.

Finally, our analysis found that CPs were the main initiators of collaborative practice, with perhaps more to gain from closer working.^63^ CPs as relationship initiators are a consistent factor in other studies as well.^11^ One explanation could be a power disparity evident in many primary care settings. While the influence this asymmetry in power has on communication and trust building was missing from earlier models of collaborative working relationships, it has been considered in other research,^43,48^ including a similar systematic review in 2019.^13^Addressing this imbalance is likely to require coordinated support from professional societies, educational institutions, and policymakers.

### Implications for research and practice

Many countries are facing ageing populations and change is vital to ensure healthcare systems can cope with the double burden of increasing chronic diseases and workforce shortages.^64^ Collaborative partnerships between GPs and CPs have the potential to mitigate these issues. Strategies to enhance trust and mutual understanding between both professions should focus not only on developing and sustaining the GP-CP relationship, but also on addressing the unequal motivations to collaborate, by closer attention to relative costs and benefits.

Further research, including experimental (trial) designs, in different settings will help policymakers to prioritise actions for sustained, successful collaborative practices.

## Trial registration number

PROSPERO registration CRD42023418394.

## Data

The dataset relied on in this article is available from the corresponding author on reasonable request.
